# Polygenic Risk for Major Depression Interacts with Parental Criticism in Predicting Adolescent Depressive Symptom Development

**DOI:** 10.1007/s10964-020-01353-4

**Published:** 2020-11-23

**Authors:** Stefanie A. Nelemans, Marco Boks, Bochao Lin, Tineke Oldehinkel, Pol van Lier, Susan Branje, Wim Meeus

**Affiliations:** 1 Department of Youth and Family, Utrecht University, Utrecht, The Netherlands; 2 Department of Psychiatry, University Medical Center Utrecht, Utrecht, The Netherlands; 3 Department of Translational Neuroscience, Brain Center Rudolf Magnus, Utrecht, The Netherlands; 4 Department of Psychiatry, University Medical Center Groningen, Groningen, The Netherlands; 5 Department of Developmental Psychology, VU University Amsterdam, Amsterdam, The Netherlands; 6 Department of Developmental Psychology, Tilburg University, Tilburg, The Netherlands

**Keywords:** Depressive symptoms, Adolescence, Longitudinal, Polygenic risk score (PRS), Parenting, Gene-by-environment interaction (G × E)

## Abstract

Research has focused more and more on the interplay between genetics and environment in predicting different forms of psychopathology, including depressive symptoms. While the polygenic nature of depressive symptoms is increasingly recognized, only few studies have applied a polygenic approach in gene-by-environment interaction (G × E) studies. Furthermore, longitudinal G × E studies on developmental psychopathological properties of depression are scarce. Therefore, this 6-year longitudinal community study examined the interaction between genetic risk for major depression and a multi-informant longitudinal index of critical parenting in relation to depressive symptom development from early to late adolescence. The sample consisted of 327 Dutch adolescents of European descent (56% boys; *M*_age_ T_1_ = 13.00, *SD*_age_ T_1_ = 0.44). Polygenic risk for major depression was based on the Hyde et al. (*Nature Genetics*, *48*, 1031–1036, 2016) meta-analysis and genetic sensitivity analyses were based on the 23andMe discovery dataset. Latent Growth Models suggested that polygenic risk score for major depression was associated with higher depressive symptoms across adolescence (significant main effect), particularly for those experiencing elevated levels of critical parenting (significant G × E). These findings highlight how polygenic risk for major depression in combination with a general environmental factor impacts depressive symptom development from early to late adolescence.

## Introduction

Major Depressive Disorder (MDD; American Psychiatric Association 2013) is a common (Kessler et al. 2007) and persistent disorder (Eaton et al. 2008) that is associated with a variety of comorbid mental health problems and impaired functioning in a wide range of domains (Maske et al. 2016). A clinical diagnosis of MDD represents the extreme end of a continuous distribution of symptom severity at the population level (Widiger and Samuel 2005). The same symptoms that define MDD are variable in the general population (Hankin et al. 2005; Liu 2016) and adolescence is a critical period for the development of such symptoms (Kessler et al. 2012). Consequently, research that focuses on the development of depressive symptoms in adolescence and identifies factors that affect this development is essential. While research has focused increasingly on the interplay between genetics and environment, studies in adolescence are scarce and few have used a longitudinal design to consider the developmental psychopathological properties of depression. Moreover, only few studies have applied a polygenic approach in gene-by-environment interaction (G × E) studies. Therefore, this longitudinal community study examined the interaction between polygenic risk for major depression and critical parenting in relation to depressive symptom development from early to late adolescence.

The etiology of depression is complex and results from both genetic and environmental factors (Garber and Rao 2014). Concerning genetic factors, twin and family studies have indicated that MDD is a moderately heritable trait (Sullivan et al. 2000). Despite robust evidence for heritability of MDD, unraveling the genetic architecture of complex traits such as MDD and identifying specific “vulnerability genes” has proven quite a challenge. Recent progress in molecular genetic analyses has led to the critical insight that the genetic basis for complex traits such as MDD is polygenic (Purcell et al. 2009), that is, resulting from the additive effect of many genetic variants (single-nucleotide polymorphisms; SNPs) with small effect sizes each. This has caused the field to move from investigating simple, single genetic markers (i.e., a so-called *candidate gene approach*) to more complex genetic indices based on multiple genes (i.e., a so-called *polygenic approach*; Belsky and Israel 2014; Wray et al. 2014). Moreover, whereas initial genetic main effect research was strongly hypothesis-driven and biologically based by focusing on genetic variation associated with specific biological functions (e.g., genetic variation associated with neurotransmitters dopamine and serotonin), research in the field has also become more hypothesis-free and data-driven (or data-inferred) in genome-wide association studies. Concerning the latter, a recent large-scale meta-analysis was among the first to identify 17 independent SNPs reaching genome-wide significance (*p* < 5 × 10^−8^) that were robustly associated with increased risk of self-reported major depression in adults of European descent (Hyde et al. 2016). Because major depression represents the extreme end of a continuous distribution of depressive symptoms at the population level (Widiger and Samuel 2005), this polygenic risk score for major depression may also be relevant for adolescent depressive symptom development. However, it is still unknown to this moment whether and how polygenic risk for major depression as identified among adults is associated with the development of depressive symptoms in adolescents from the general population. Hence, the first goal of the present study was to rely on findings from the Hyde et al. (2016) meta-analysis to examine the association between polygenic risk for major depression and depressive symptom development in an adolescent community sample. Because sex differences in depression have been found to emerge in adolescence (Hankin et al. 2015), it was also explored whether girls could be more genetically vulnerable in a way that genetic risk for major depression is more strongly associated with adolescent depressive symptom development for girls compared to boys (Merikangas and Almasy 2020; in part, for example, through affected hormonal processes; Naninck et al. 2011).

In addition to genetic factors, environmental factors are assumed to play a prominent role in the development of depressive symptoms in adolescence (Garber and Rao 2014). An important environmental factor in adolescent depressive symptom development is rejecting and particularly aversive parenting, which has been modestly but systematically associated with higher levels of child and adolescent depressive symptoms across studies (for a meta-analysis, see McLeod et al. 2007). Parental criticism, which refers to negative comments expressed by parents to their child and reflects a non-supportive and critical emotional family climate, may be specifically important to consider in this respect. Historically, a critical family climate, which is at the core of Brown’s Expressed Emotion theory (Brown et al. 1972; further refined by Vaughn and Leff 1976), has been associated with relapse in depressed patients (Hooley 2007). More recently, parental criticism had received increased attention in association with the development of depressive symptoms among youth (Peris and Miklowitz 2015), also since the importance of (perceived) parental criticism on youth psychological adjustment has become central in other prominent theories, such as Parental Acceptance-Rejection Theory (PARTheory; Rohner et al. 2005). Importantly, studies have indeed shown that higher levels of parental criticism are associated with higher levels of adolescent depressive symptoms, both cross-sectionally and longitudinally (e.g., Nelemans et al. 2014). It has been suggested that adolescents may internalize parental criticism as self-criticism or a negative evaluation of the self and significant others, which in turn is associated with higher depressive symptoms (e.g., Bolton et al. 2009; Rohner et al. 2005).

However, not all adolescents exposed to parental criticism develop depressive symptoms to the same extent, which raises questions about individual differences in vulnerability to adverse environments. Importantly, research on gene-by-environment interactions (G × E) builds on the assumption that people vary in the extent to which they are affected by environmental factors and that this sensitivity to the environment may be genetically predisposed (Pluess 2015). Historically, the two most prominent theories on environmental sensitivity are the Diathesis-Stress (or Dual-Risk) framework and the Differential Susceptibility framework (which are currently integrated in single overarching meta-framework of environmental sensitivity; Pluess 2015). On the one hand, in the Diathesis-Stress framework (Monroe and Simons 1991) environmental sensitivity is seen primarily as vulnerability for developing problematic outcomes in individuals faced with environmental adversity. In the context of this study, the negative effect of parental criticism on adolescent depressive symptom development would be expected to be greatest for youth with stronger polygenic risk for major depression (i.e., genetically vulnerable youth). On the other hand, in the Differential Susceptibility framework (Belsky et al. 2007; Belsky and Pluess 2009) environmental sensitivity is seen as susceptibility with some being more and some less susceptible to both negative and positive environmental influences (*for better and for worse*).

Following pioneering G × E work on MDD (Caspi et al. 2003), several studies have supported interactions between individual genetic differences and exposure to adverse environments, such as chronic stress (e.g., Hammen et al. 2010), stressful or negative life events (e.g., Chen et al. 2013), and maladaptive parenting (e.g., Van Assche et al. 2017), in predicting depressive symptoms in youth (for a systematic review, see Dunn et al. 2011). Most of these studies were hypothesis-driven and investigated only a limited number of genetic variants in relevant biological pathways and findings generally suggested that adverse environments are particularly associated with higher depressive symptoms for youth with a genetic predisposition (in line with the Diathesis-Stress framework; Monroe and Simons 1991). However, also many hypothesis-driven G × E studies have failed to replicate these findings (Dunn et al. 2011; Van der Auwera et al. 2018). Critical reflections on these hypothesis-driven G × E studies have resulted in recommendations for future research to include a polygenic approach (Dick et al. 2015), given that complex traits such as major depression appear to result from the additive effect of many genetic variants with small effect sizes individually. The second goal of the present study was therefore to investigate whether the effect of parental criticism, as indicator of an adverse parental environment, on adolescent depressive symptom development was moderated by polygenic risk for major depression. Given the emergence of sex differences in depression in adolescence (Hankin et al. 2015) and some indications for G × E in relation to depressive symptoms for girls but not boys (Hammen et al. 2010), sex differences in G × E predicting adolescent depressive symptom development were also explored. Adolescent girls show stronger sensitivity to interpersonal stress, in a way that they experience negative interpersonal events, such as parental criticism, as more stressful than boys, and this has been associated with gender differences in adolescent depressive symptom development (Rudolph 2009). It was therefore explored whether the hypothesized stronger negative effect of parental criticism on adolescent depressive symptom development for genetically vulnerable youth would be stronger for girls compared to boys.

## Current Study

The present 6-year longitudinal community study aimed to examine (1) whether data-inferred polygenic risk for major depression (as identified among adults of European descent in a recent meta-analysis; Hyde et al. 2016) was associated with adolescent depressive symptom development from early to late adolescence, and (2) whether data-inferred polygenic risk for major depression moderated the effect of parental criticism on adolescent depressive symptom development from early to late adolescence. Concerning the main effect of data-inferred polygenic risk on adolescent depressive symptom development, in line with a developmental psychopathological perspective (e.g., Cicchetti and Rogosch 2002) it was hypothesized that polygenic risk for major depression as identified among adults (Hyde et al. 2016) would already express itself in adolescence in the form of higher initial depressive symptoms at the start of adolescence and/or increasing depressive symptoms across adolescence. In addition, potential sex differences were explored in the association between genetic risk for major depression and adolescent depressive symptom development. Concerning the effect of G × E on adolescent depressive symptom development, in line with the Diathesis-Stress framework (Monroe and Simons 1991) it was hypothesized that parental criticism would be particularly associated with higher initial depressive symptoms at the start of adolescence and/or increasing depressive symptoms across adolescence for adolescents with higher polygenic risk for major depression. In this way, the negative effect of parental criticism on adolescent depressive symptom development would be expected to be greatest for genetically vulnerable youth. In addition, sex differences in G × E predicting adolescent depressive symptom development were explored. For the main analyses, the data-inferred polygenic risk score was based on the 17 genetic variants across the genome that were most strongly associated with major depression (*p* < 5 × 10^−8^) in a recent meta-analysis (Hyde et al. 2016). Moreover, this study included several important genetic sensitivity analyses. For these sensitivity analyses, information from the 23andMe discovery dataset was used to calculate 12 additional data-inferred polygenic risk scores, each based on more genetic variants with increasingly weaker associations with major depression but thereby better reflecting the total SNP-heritability^1^ of major depression (for more information, please see the section on “Polygenic Risk Scores for Major Depression” in “Methods” below).

## Methods

### Participants

Participants in this 6-year longitudinal community study were 369 adolescents (56.6% boys; *M*_age_ T_1_ = 13.00, *SD*_age_ T_1_ = 0.44; 7.1% from low SES families based on parents’ job level), who provided saliva samples to extract DNA, and their mothers (*M*_age_ T_1_ = 44.65, *SD*_age_ T_1_ = 4.44). Participants were part of the ongoing Research on Adolescent Development And Relationships (RADAR—young cohort; 10.17026/dans-zrb-v5wp) study and identified themselves as ethnic Dutch. All participants attended the first grade of secondary school at the start of the study. Following quality control of the genetic data, including exclusion of ethnic outliers, the study sample consisted of 327 adolescents (56.3% boys; *M*_age_ T_1_ = 13.00, *SD*_age_ T_1_ = 0.44; 6.5% from low SES families based on parents’ job level) and their mothers (*M*_age_ T_1_ = 44.72, *SD*_age_ T_1_ = 4.25).^2^ There were no significant differences between participants in the total genetic sample (*N* = 369) and the study sample with high-quality genetic data (*n* = 327) concerning sex, χ²(1) = 0.16, *p* = 0.69, SES, χ²(1) = 1.62, *p* = 0.20, age, *F*(1, 367) = 0.25, *p* = 0.62, adolescent depressive symptoms T_1,_ *F*(1, 363) = 0.74, *p* = 0.39, adolescent-reported parental criticism T_1_, *F*(1, 355) = 0.60, *p* = 0.44, and mother-reported criticism T_1_, *F*(1, 366) = 1.16, *p* = 0.28.

Sample attrition was low over time, with 312 of the 327 adolescents (4.6% attrition from wave 1 to wave 6) and 309 of the 327 mothers (5.5% attrition from wave 1 to wave 6) still participating at the sixth annual measurement wave. There were no significant differences between adolescents participating at all six measurement waves and those dropping out of the study concerning sex, χ²(1) = 0.59, *p* = 0.44, SES, χ²(1) = 1.10, *p* = 0.30, age, *F*(1, 325) = 0.01, *p* = 0.93, adolescent depressive symptoms T_1,_ *F*(1, 321) = 1.43, *p* = 0.23, adolescent-reported parental criticism T_1_, *F*(1, 317) = 0.13, *p* = 0.72, and mother-reported criticism T_1_, *F*(1, 324) = 1.26, *p* = 0.26. No genetic data were missing for the study sample (*n* = 327). Most missing data on the variables of interest were the result of dropout, with some occasional missings being due to unavailability of participants at a specific measurement wave or participants’ decision not to complete certain parts of the questionnaire at a specific measurement wave. Specifically, missing data on adolescent depressive symptoms ranged between 1.22 and 4.89% across waves, missing data on adolescent-reported parental criticism ranged between 2.45 and 6.12% across waves, and missing data on mother-reported criticism ranged between 0 and 5.81% across waves. Little’s Missing Completely at Random (MCAR) test showed that data were missing at random, χ^2^(452) *=* 475.09, *p* = 0.22. Missing data were handled in M*plus* with Full Information Maximum Likelihood (Muthén and Muthén 1998).

### Procedure

Participants were recruited from randomly selected primary schools in the western and central regions of the Netherlands (Western Europe). All participants and their parents received a complete description of the study and provided active written informed consent before the start of the study (*N* = 497). For six successive years, adolescents and their mothers completed annual questionnaires during a home visit and received a small monetary compensation for every wave they completed the questionnaires. In addition, at the fifth measurement wave both parents and adolescents were again asked for active consent to collect saliva samples from adolescents to obtain DNA. In total, 369 adolescents provided consent to provide a saliva sample to extract DNA, for which these participants received an additional small monetary compensation. The RADAR study was approved by the board of the local research institute and by the Medical Ethical Committees of Utrecht University and VU University Amsterdam.

### DNA Processing and Genetic Profiling

Genotyping of all participants was done with the Affymetrix 6.0 array (McCarroll et al. 2008) using DNA from saliva samples. The genotype calling was performed with Birdseed 2 algorithm (Korn et al. 2008; McCarroll et al. 2008) with the Affymetrix 3.3 APT software on all samples simultaneously. Samples were removed if the Affymetrix CQC < 0.40, the genotyping calling rate < 0.90, the heterozygosity *F* value was < 0.10 or > 0.10, or the DNA gender of the sample did not match the phenotype gender. Samples were also removed if the 10 genetic principal components indicated a Caucasian European (CEU) ethnic outlier after projection of the study samples on the 1000 Genomes reference sample. Single nucleotide polymorphisms (SNPs) were filtered using Plink 1.07 (Purcell et al. 2007) based on the following criteria: No or incorrect mapping on Build 37 HG19 of the human genome, inconsistent calls in plate control samples with an error rate > 1%, < 0.95 genotyping rate, minor allele frequencies (MAF) < 0.01, Hardy–Weinberg equilibrium *p* < 0.000001. After this quality control (QC), all SNPs were strand aligned to the 1000 Genomes phase 1 integrated release version 3 panel (Altshuler et al. 2012). Subsequently, the genotype data were phased using SHAPEIT2 (Delaneau et al. 2013). All the samples were genotyped by the Affymetrix 6.0 array, with a high variant calling rate (> 0.96%; *N* of genotyped SNPs in the samples ranged from 650,005 to 674,172). Imputation was performed from the HRC and 1000 Genomes Project Phase 3 (v5) reference panels using a two-stage approach. Pre-imputation phasing and imputation of genotype platform specific SNPs was carried out using the MACH software (Li et al. 2010). Subsequently, imputation of the reference set was carried out with Minimac. There were 16,619,494 SNPs after imputation for further post-imputation quality control.

After imputation, SNPs that failed the following criteria were excluded: MAF > 0.01, MAF and the reference allele frequency difference < 0.20, and Hardy–Weinberg equilibrium > 0.00001, Mendelian error < 0.02. This resulted in 10,837,431 remaining SNPs. Furthermore, SNPs with imputation values above 0.8 and a MAF > 0.01 in both the discovery dataset and the target dataset were extracted (*n* = 7,585,001) and clumped using PLINK 1.90 (Chang et al. 2015) in two rounds to remove high linkage disequilibrium (LD) regions and avoid LD inflating the polygenic risk scores: (1) physical distance threshold 250 kb and LD threshold R^2^ of 0.5, and (2) physical distance threshold 5000 kb and LD threshold R^2^ of 0.2 (McLaughlin et al. 2017; Pries et al. 2020). Lastly, complex LD regions were removed including the Major HistoCompatibility (MHC) region. In the end, 571,857 LD pruned SNPs remained for the polygenic risk score calculation.

### Polygenic Risk Scores for Major Depression

For the main analyses, this study used the genome-wide association study summary statistics (i.e., the beta-values, effective alleles, and *p* values) from the a recent meta-analysis in individuals of European descent (Hyde et al. 2016) to calculate a data-inferred polygenic risk score for MDD based on the exact same 17 independent SNPs that reached genome-wide significance (*p* < 5 × 10^−8^) in their joint analysis of the 23andMe discovery dataset, the Psychiatric Genomics Consortium dataset (including the Psychiatric Genomics Consortium MDD, bipolar disorder, SCZ1, SCZ1 + SWE, and the SCZ2 datasets), and the 23andMe replication dataset. Since this calculated polygenic risk score only included information on those 17 SNPs that reached genome-wide significance (*p* < 5 × 10^−8^) in this meta-analysis, this score reflected the additive genetic risk of only those few genetic variants that were found to be most strongly associated with major depression. However, the maximum proportion of variation in major depression that can be explained by additive effects of SNPs is captured by many more additional genetic variants that are less strongly associated with major depression than *p* < 5 × 10^−8^.

For the sensitivity analyses, the full genome-wide association study summary statistics were therefore requested for the 23andMe discovery dataset^3^ (i.e., the beta-values, effective alleles, and *p* values) to calculate 12 additional data-inferred polygenic risk scores for major depression in this study. Specifically, each of these scores included information on more and more genetic variants with increasingly weaker associations with major depression and thereby better reflected the total SNP-heritability of major depression. The following different *p-*value thresholds for the additional data-inferred polygenic risk scores were used: 5 × 10^−7^ (48 SNPs), 5 × 10^−6^ (136 SNPs), 5 × 10^−5^ (410 SNPs), 5 × 10^−4^ (1,771 SNPs), 5 × 10^−3^ (9,428 SNPs), 0.01 (15,779 SNPs), 0.05 (55,013 SNPs), 0.10 (95,470 SNPs), 0.20 (166,109 SNPs), 0.30 (229,496 SNPs), 0.40 (288,036 SNPs) and 0.50 (342,808 SNPs). This data-driven approach in which polygenic risk scores are based on different *p* value thresholds is common practice and recommended (Belsky and Israel 2014; Choi et al. 2020), particularly in cases of highly polygenic traits for which most genetic effects are very small. In such cases, more lenient *p*-value thresholds and thus more genetically-inclusive polygenic risk scores may better capture the total genetic risk associated with a certain phenotype (SNP-heritability). Importantly, the *p*-value thresholds used to create polygenic risk scores are very different from *p* < 0.05 in null hypothesis significance testing: Whereas polygenic risk scores based on only those SNPs that reached genome-wide significance (*p* < 5 × 10^−8^) or other strict *p-*value thresholds (e.g., *p* < 5 × 10^−5^) capture the additive effect of the genetic variants most strongly associated with a certain phenotype, a *p-*value threshold of 1.00 is expected to capture the maximum proportion of SNP heritability in a certain phenotype. From this point forward, the term “genetically-selective” polygenic risk scores is used to refer to the data-inferred polygenic risk scores based on stricter *p*-value thresholds (e.g., *p* < 5 × 10^−7^, *p* < 5 × 10^−5^) and “genetically-inclusive” polygenic risk scores to refer to the data-inferred polygenic risk scores based on more lenient *p* value thresholds (e.g., *p* < 0.01, *p* < 0.30). All data-inferred polygenic risk scores were created using PLINK version 1.90. Just like in the recent meta-analysis (Hyde et al. 2016) this study built upon, all data-inferred polygenic risk scores were corrected for population stratification (i.e., the top five Dutch principal components resulting from PLINK’s Principal Components Analysis,^4^ Abdellaoui et al. 2014, as recommended by Tucker et al. 2014), adolescents’ age, and adolescents’ sex before being included in any of the subsequent analyses.

### Measures

#### Depressive symptoms

The Reynolds Adolescent Depression Scale, second edition (RADS-2; Reynolds 2000) was used to assess adolescent depressive symptoms. The RADS-2 is a self-report questionnaire that consists of 23 items, which are measured on a 4-point scale ranging from 1 (*almost never*) to 4 (*usually*). Sample items include “I am sad” and “I feel like crying.” In this study, internal consistency for the total depression scale was found to be good across waves, with Cronbach’s alpha ranging between 0.93 and 0.95. Previous studies have shown good psychometric properties for the RADS-2 among adolescents (Osman et al. 2010).

#### Parental criticism

The 5-item parental criticism subscale of the 38-item Level of Expressed Emotions Scale (LEE; Hale et al. 2007) was used to assess parental criticism as reported by adolescents and their mothers. Specifically, adolescents reported on their perceived criticism from both parents, while mothers reported on their own criticism towards their adolescent. Items were rated on a 4-point scale, ranging from 1 (*untrue*) to 4 (*true*). Sample items include “My parents are critical of me/I am critical of my child” and “My parents try to change me/I try to change my child”. This study combined adolescent- and mother-reports of parental criticism across six successive years into a single multi-informant longitudinal average, with higher levels representing higher levels of parental criticism. Cronbach’s alpha internal consistency for this multi-informant score was good across waves, ranging between 0.69 and 0.77. Previous studies have shown good psychometric properties for the 38-item LEE (Hale et al. 2007).

### Statistical Analyses

Latent Growth Curve Models (LGCMs) in M*plus* Version 8.3 (Muthén and Muthén 1998–2017) were constructed to capture initial levels of depressive symptoms at the first wave in early adolescence (i.e., the intercept) and change in these symptoms across 6 successive years (i.e., the linear and quadratic slopes). As a first step, the corrected polygenic risk scores for major depression were included as predictor of the intercept and slopes of adolescent depressive symptoms, whilst controlling for sex differences in adolescent depressive symptom development.^5^ As a second step, the multi-informant longitudinal index of parental criticism and the interaction between parental criticism and the polygenic risk scores for major depression were additionally included as predictors. Both the polygenic risk scores for major depression and multi-informant longitudinal index of parental criticism were standardized before creating the interaction terms. In this second step, the correlation between the polygenic risk scores for major depression and the multi-informant longitudinal index of parental criticism was also included to account for potential gene-environment correlation. Significant interactions between parental criticism and the polygenic risk scores for major depression were interpreted by visualizing predicted intercept or slope values of adolescent depressive symptoms at low (−1 SD) and high (+1 SD) levels of parental criticism and polygenic risk for major depression.

In all LGCMs, ML estimation with standard errors and chi-square robust to non-normality was used (i.e., MLR estimator; Muthén and Muthén 1998–2017). Model fit was assessed with the Comparative Fit Index (CFI), the Root Mean Squared Error of Approximation (RMSEA) and its 90% confidence interval (CI), and the Standardized Root Mean Square Residual (SRMR), using conventional standards (Hu and Bentler 1999; Kline 2005).

## Results

### Descriptive Statistics

A summary of the most relevant correlations among all study variables can be found in Table 1. Correlations between the corrected polygenic risk scores for major depression and adolescent depressive symptoms across time were in most instances significant but small in effect size, *r*s = 0.07–0.20, just like the effect size of correlations between the corrected polygenic risk scores for major depression and parental criticism, *r*s = 0.05–0.21. In addition, correlations between and adolescent depressive symptoms across time were significant and medium in effect size, *r*s = 0.37–0.43. Means and standard deviations of all variables in the statistical analyses can be found in Table 2.

**Table 1:** Summary of correlations among the different polygenic risk scores and all study variables (*N* = 327)

Variable	1	2	3	4	5	6	7
1. Depressive symptoms T_1_	–						
2. Depressive symptoms T_2_	0.58***	–					
3. Depressive symptoms T_3_	0.56***	0.66***	–				
4. Depressive symptoms T_4_	0.55***	0.64***	0.71***	–			
5. Depressive symptoms T_5_	0.51***	0.57***	0.64***	0.79***	–		
6. Depressive symptoms T_6_	0.51***	0.53***	0.61***	0.70***	0.75***	–	
7. Parental criticism^a^	0.37***	0.39***	0.38***	0.43***	0.42***	0.37***	–
8. PRS MDD 5 × 10^−8b^	0.15**	0.13*	0.16**	0.17**	0.13*	0.11*	0.05
9. PRS MDD 5 × 10^−7^	0.10	0.18***	0.18**	0.19***	0.19***	0.14*	0.09
10. PRS MDD 5 × 10^−6^	0.07	0.17**	0.16**	0.17**	0.17**	0.12*	0.06
11. PRS MDD 5 × 10^−5^	0.11*	0.12*	0.15**	0.18***	0.20***	0.14*	0.07
12. PRS MDD 5 × 10^−4^	0.12*	0.11*	0.13*	0.16**	0.16**	0.14**	0.06
13. PRS MDD 5 × 10^−3^	0.10	0.15**	0.15**	0.13*	0.11*	0.13*	0.08
14. PRS MDD 0.01	0.10	0.14**	0.15**	0.13*	0.13*	0.16**	0.09
15. PRS MDD 0.05	0.13*	0.18**	0.19***	0.16**	0.15**	0.17**	0.15**
16. PRS MDD 0.10	0.13*	0.18**	0.18**	0.15**	0.14*	0.17**	0.14**
17. PRS MDD 0.20	0.14*	0.19***	0.18**	0.17**	0.16**	0.18**	0.18**
18. PRS MDD 0.30	0.14*	0.18***	0.18**	0.16**	0.15**	0.18**	0.19***
19. PRS MDD 0.40	0.15**	0.19***	0.19***	0.17**	0.16**	0.18**	0.21***
20. PRS MDD 0.50	0.15**	0.19***	0.19***	0.17**	0.16**	0.18**	0.20***

PRS MDD […] = polygenic risk score for major depression, calculated based on the mentioned *p-*value threshold

^a^Adolescent- and mother-reports of parental criticism across 6 successive years were combined into a single multi-informant longitudinal average, with higher levels representing higher levels of parental criticism

^b^The PRS MDD 5 × 10^−8^ score was based on the meta-analysis by Hyde et al. (2016) and corrected for adolescent age, sex, and population stratification. The other 12 polygenic risk scores for major depression with different *p-*value thresholds were based on the 23andMe summary statistics and also corrected for adolescent age, sex, and population stratification

^*^p < 0.05; ^**^p < 0.01; ^***^p < 0.001

**Table 2:** Means and standard deviations of all variables included in the statistical analyses (*N* = 327)

	*M*	*SD*
Depressive symptoms T_1_	1.62	0.49
Depressive symptoms T_2_	1.49	0.49
Depressive symptoms T_3_	1.52	0.53
Depressive symptoms T_4_	1.55	0.54
Depressive symptoms T_5_	1.53	0.53
Depressive symptoms T_6_	1.59	0.56
Adolescent-reported parental criticism T_1_	1.64	0.47
Adolescent-reported parental criticism T_2_	1.67	0.51
Adolescent-reported parental criticism T_3_	1.76	0.57
Adolescent-reported parental criticism T_4_	1.80	0.56
Adolescent-reported parental criticism T_5_	1.77	0.57
Adolescent-reported parental criticism T_6_	1.77	0.57
Mother-reported parental criticism T_1_	1.57	0.39
Mother-reported parental criticism T_2_	1.54	0.38
Mother-reported parental criticism T_3_	1.52	0.38
Mother-reported parental criticism T_4_	1.54	0.38
Mother-reported parental criticism T_5_	1.54	0.39
Mother-reported parental criticism T_6_	1.52	0.38
Parental criticism^a^	1.63	0.32
PRS MDD 5 × 10^−8b^	−8.75 × 10^−6^	2.87 × 10^−3^
PRS MDD 5 × 10^−7^	−3.92 × 10^−5^	2.38 × 10^−3^
PRS MDD 5 × 10^−6^	−1.56 × 10^−5^	1.35 × 10^−3^
PRS MDD 5 × 10^−5^	−1.67 × 10^−5^	7.15 × 10^−4^
PRS MDD 5 × 10^−4^	−4.88 × 10^−6^	2.88 × 10^−4^
PRS MDD 5 × 10^−3^	−7.41 × 10^−8^	1.14 × 10^−4^
PRS MDD 0.01	6.84 × 10^−7^	8.78 × 10^−5^
PRS MDD 0.05	1.28 × 10^−6^	4.41 × 10^−5^
PRS MDD 0.10	1.12 × 10^−6^	3.16 × 10^−5^
PRS MDD 0.20	9.45 × 10^−7^	2.20 × 10^−5^
PRS MDD 0.30	7.19 × 10^−7^	1.75 × 10^−5^
PRS MDD 0.40	6.32 × 10^−7^	1.48 × 10^−5^
PRS MDD 0.50	5.52 × 10^−7^	1.28 × 10^−5^

PRS MDD […] = polygenic risk score for major depression, calculated based on the mentioned *p-*value threshold

^a^Adolescent- and mother-reports of parental criticism across six successive years were combined into a single multi-informant longitudinal average, with higher levels representing higher levels of parental criticism. For the analyses, this score was standardized to facilitate estimation and interpretation

^b^The PRS MDD 5 × 10^−8^ score was based on the meta-analysis by Hyde et al. (2016) and corrected for adolescent age, sex, and population stratification. The other 12 PRS MDD scores with different *p-*value thresholds were based on the 23andMe summary statistics and also corrected for adolescent age, sex, and population stratification. For the analyses, all PRS MDD were standardized to facilitate estimation and interpretation

### Adolescent Depressive Symptom Development

**Fig. 1:**
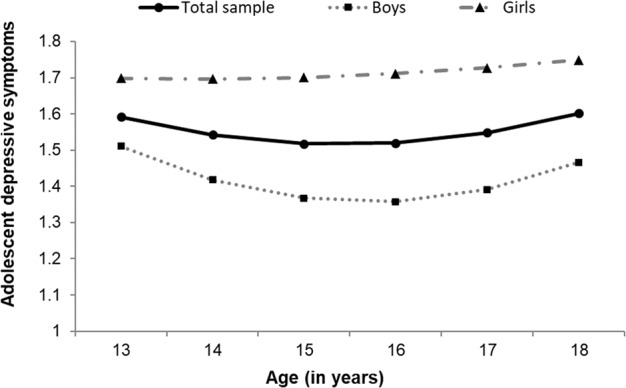
Graphical representation of adolescent depressive symptom development from early to late adolescence across six successive waves (i.e., approximately ages 13–18 years) for the total sample (*N* = 327), as well as boys (*n* = 184) and girls (*n* = 143) separately

Fit of the LGCM of adolescent depressive symptoms without any predictors was good, χ^2^(12) = 20.66, RMSEA [90% CI] = 0.05 [0.00, 0.08], CFI = 0.99, SRMR = 0.03. In the total sample, adolescent depressive symptoms showed an initial slight decrease, *b*_linear slope_ = −0.06, *p* < 0.001, followed by a slight increase over time, *b*_quadratic slope_ = 0.01, *p* < 0.001 (see Fig. 1). In all main models, significant main effects of sex on adolescent depressive symptom development were found. Specifically, girls showed higher intercept levels of adolescent depressive symptoms, βs = 0.47–0.55, *p*s < 0.001, and a less steep initial decrease in depressive symptoms, βs_linear slope_ = 0.53–0.58, *p*s = 0.002–0.005, followed by a less steep increase in depressive symptoms over time, βs_quadratic slope_ = −0.49–−0.54, *p*s = 0.006–0.011. This suggests higher mean levels and stronger stability in depressive symptoms across time for adolescent girls compared to adolescent boys (see Fig. 1).

### Polygenic Risk for Major Depression and Adolescent Depressive Symptom Development

Fit of the models including the corrected polygenic risk scores for major depression and adolescent sex as predictors of the intercept and both linear and quadratic growth of adolescent depressive symptoms was good, χ^2^(18) ≤ 29.47, RMSEA [90% CI] ≤ 0.04 [0.01,0.07], CFI = 0.99, SRMR = 0.03. Importantly, in line with expectations, higher polygenic risk (i.e., stronger genetic risk for major depression; as identified by Hyde et al. 2016) was significantly associated with higher intercept levels of adolescent depressive symptoms, β = 0.17, *p* = 0.019. Moreover, the sensitivity analyses suggested replication of this finding across nearly all 12 additional polygenic risk scores; higher levels of polygenic risk for major depression were consistently significantly associated with higher intercept levels of adolescent depressive symptoms, βs = 0.09–0.21, *p*s = 0.001–0.159 (see Table 3). Polygenic risk for major depression was not (consistently) significantly associated with changes in adolescent depressive symptoms over time (i.e., the linear or the quadratic slope).

**Table 3:** Summary of standardized coefficients of polygenic risk for MDD predicting adolescent depressive symptom development, controlling for sex (*N* = 327)

Model	Intercept^a^	Linear slope	Quadratic slope
PRS MDD 5 × 10^−8b^	β = **0.17**, *p* = **0.019**	β = 0.08, *p* = 0.412	β = −0.10, *p* = 0.324
PRS MDD 5 × 10^−7^	β = **0.13**, *p* = **0.038**	β = **0.19**, *p* = **0.022**	β = −**0.18**, *p* = **0.041**
PRS MDD 5 × 10^−6^	β = 0.09, *p* = 0.159	β = **0.21**, *p* = **0.018**	β = −**0.20**, *p* = **0.034**
PRS MDD 5 × 10^−5^	β = 0.12, *p* = 0.083	β = 0.15, *p* = 0.127	β = −0.12, *p* = 0.236
PRS MDD 5 × 10^−4^	β = **0.13**, *p* = **0.043**	β = 0.08, *p* = 0.435	β = −0.05, *p* = 0.631
PRS MDD 5 × 10^−3^	β = **0.14**, *p* = **0.017**	β = 0.08, *p* = 0.435	β = −0.07, *p* = 0.509
PRS MDD 0.01	β = **0.16**, *p* = **0.011**	β = 0.04, *p* = 0.696	β = −0.01, *p* = 0.946
PRS MDD 0.05	β = **0.19**, *p* = **0.002**	β = 0.07, *p* = 0.542	β = −0.05, *p* = 0.656
PRS MDD 0.10	β = **0.19**, *p* = **0.003**	β = 0.04, *p* = 0.711	β = −0.02, *p* = 0.842
PRS MDD 0.20	β = **0.20**, *p* = **0.002**	β = 0.05, *p* = 0.632	β = −0.04, *p* = 0.744
PRS MDD 0.30	β = **0.20**, *p* = **0.001**	β = 0.04, *p* = 0.701	β = −0.03, *p* = 0.812
PRS MDD 0.40	β = **0.21**, *p* = **0.001**	β = 0.06, *p* = 0.595	β = −0.05, *p* = 0.685
PRS MDD 0.50	β = **0.21**, *p* = **0.001**	β = 0.05, *p* = 0.654	β = −0.04, *p* = 0.729

PRS MDD […] = polygenic risk score for major depression, calculated based on the mentioned *p-*value threshold. Significant main effects (*p* < 0.05) are in bold

^a^The average age of participants at the intercept was 13.00 years old (*SD* = 0.44)

^b^The PRS MDD 5 × 10^−8^ score was based on the meta-analysis by Hyde et al. (2016) and corrected for adolescent age, sex, and population stratification. The other 12 polygenic risk scores for major depression with different *p-*value thresholds were based on the 23andMe summary statistics and also corrected for adolescent age, sex, and population stratification

Fit of the models including the exploratory sex × polygenic risk for major depression interactions was good, χ^2^(21) ≤ 33.00, RMSEA [90% CI] ≤ 0.04 [0.01, 0.07], CFI = 0.99, SRMR = 0.03. The exploratory sex × polygenic risk for major depression interactions were not consistently significantly associated with intercept levels of adolescent depressive symptoms, βs = 0.07–0.24, *p*s = 0.004–0.390, nor with the initial decrease in depressive symptoms, βs_linear slope_ = 0.11–0.35, *p*s = 0.007–0.338, or the following increase in depressive symptoms over time, βs_quadratic slope_ = −0.13–−0.35, *p*s = 0.007–0.311 (see Table 4).

**Table 4:** Summary of standardized coefficients of polygenic risk for MDD × sex interactions predicting adolescent depressive symptom development (*N* = 327)

Model	Intercept^a^	Linear slope	Quadratic slope
PRS MDD 5 × 10^−8b^	β = 0.15, *p* = 0.123	β = 0.21, *p* = 0.081	β = −0.24, *p* = 0.052
PRS MDD 5 × 10^−7^	β = **0.20**, *p* = **0.011**	β = 0.15, *p* = 0.181	β = −0.14, *p* = 0.264
PRS MDD 5 × 10^−6^	β = **0.24**, *p* = **0.004**	β = 0.11, *p* = 0.338	β = −0.13, *p* = 0.311
PRS MDD 5 × 10^−5^	β = **0.24**, *p* = **0.007**	β = 0.26, *p* = 0.055	β = −0.24, *p* = 0.089
PRS MDD 5 × 10^−4^	β = 0.12, *p* = 0.185	β = **0.35**, *p* = **0.011**	β = −**0.34**, *p* = **0.015**
PRS MDD 5 × 10^−3^	β = 0.07, *p* = 0.390	β = **0.34**, *p* = **0.008**	β = −**0.35**, *p* = **0.010**
PRS MDD 0.01	β = 0.09, *p* = 0.294	β = **0.34**, *p* = **0.007**	β = −**0.35**, *p* = **0.007**
PRS MDD 0.05	β = 0.15, *p* = 0.084	β = 0.25, *p* = 0.087	β = −0.28, *p* = 0.066
PRS MDD 0.10	β = 0.14, *p* = 0.107	β = 0.22, *p* = 0.135	β = −0.25, *p* = 0.104
PRS MDD 0.20	β = 0.15, *p* = 0.098	β = 0.23, *p* = 0.119	β = −0.27, *p* = 0.077
PRS MDD 0.30	β = 0.17, *p* = 0.053	β = 0.18, *p* = 0.224	β = −0.23, *p* = 0.148
PRS MDD 0.40	β = **0.17**, *p* = **0.040**	β = 0.19, *p* = 0.194	β = −0.23, *p* = 0.141
PRS MDD 0.50	β = 0.16, *p* = 0.053	β = 0.20, *p* = 0.176	β = −0.23, *p* = 0.128

PRS MDD […] = polygenic risk score for major depression, calculated based on the mentioned *p-*value threshold. Significant interactions (*p* < 0.05) are in bold

^a^The average age of participants at the intercept was 13.00 years old (*SD* = 0.44)

^b^The PRS MDD 5 × 10^−8^ score was based on the meta-analysis by Hyde et al. (2016) and corrected for adolescent age, sex, and population stratification. The other 12 polygenic risk scores for major depression with different *p*-value thresholds were based on the 23andMe summary statistics and also corrected for adolescent age, sex, and population stratification

### Polygenic Risk for Major Depression × Parental Criticism and Adolescent Depressive Symptom Development

**Fig. 2:**
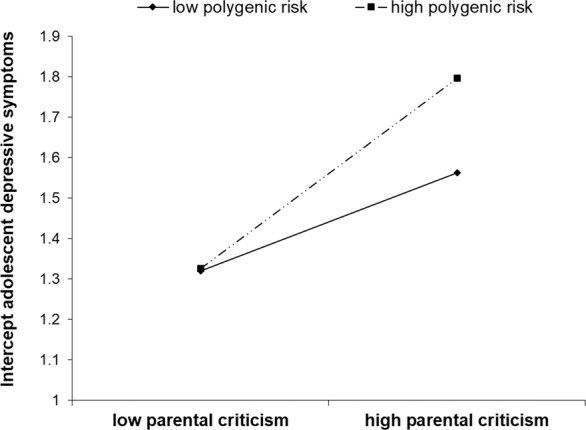
Graphical representation of the significant interaction between the corrected polygenic risk score for major depression (based on Hyde et al. 2016) and the multi-informant longitudinal index of parental criticism on intercepts levels of adolescent depressive symptoms

Fit of the models including the interaction between the corrected polygenic risk scores for major depression and the multi-informant longitudinal index of parental criticism as predictors of the intercept and both linear and quadratic growth of adolescent depressive symptoms was good, χ^2^(28) ≤ 41.62, RMSEA [90% CI] ≤ 0.04 [0.01, 0.06], CFI = 0.99–1.00, SRMR = 0.03–0.04. Importantly, in line with expectations, the interaction between the corrected polygenic risk score for major depression (i.e., genetic risk for major depression; as identified by Hyde et al. 2016) and parental criticism was significantly associated with higher intercept levels of adolescent depressive symptoms, β = 0.15, *p* = 0.008. A visualization of predicted intercept values of adolescent depressive symptoms at low (−1 SD) and high (+1 SD) levels of parental criticism and polygenic risk for major depression suggested that lower levels of parental criticism were associated with the lowest intercept levels of adolescent depressive symptoms, regardless of polygenic risk for major depression, whereas higher levels of parental criticism were associated with higher intercept levels of adolescent depressive symptoms, particularly for adolescents with higher polygenic risk for major depression (see Fig. 2). The sensitivity analyses suggested that this finding could not be replicated for most of the 12 additional polygenic risk scores, βs = −0.11–0.13, *p*s = 0.019–0.891 (see Table 5).

**Table 5:** Summary of standardized coefficients of polygenic risk for MDD × parental criticism interactions predicting adolescent depressive symptom development, controlling for sex (*N* = 327)

Model	Intercept^a^	Linear slope	Quadratic slope
PRS MDD 5 × 10^−8b^	β = **0.15**, *p* = **0.008**	β = 0.11, *p* = 0.184	β = −0.10, *p* = 0.241
PRS MDD 5 × 10^−7^	β = **0.13**, *p* = **0.019**	β = **0.20**, *p* = **0.015**	β = −**0.19**, *p* = **0.037**
PRS MDD 5 × 10^−6^	β = **0.01**, *p* = **0**.**829**	β = **0.24**, *p* = **0.007**	β = −**0.22**, *p* = **0.015**
PRS MDD 5 × 10^−5^	β = 0.01, *p* = 0.891	β = 0.20, *p* = 0.051	β = −0.14, *p* = 0.147
PRS MDD 5 × 10^−4^	β = −0.02, *p* = 0.730	β = 0.15, *p* = 0.145	β = −0.08, *p* = 0.436
PRS MDD 5 × 10^−3^	β = −0.05, *p* = 0.440	β = **0.17**, *p* = **0.048**	β = −0.15, *p* = 0.087
PRS MDD 0.01	β = −0.05, *p* = 0.452	β = 0.13, *p* = 0.131	β = −0.09, *p* = 0.302
PRS MDD 0.05	β = −0.06, *p* = 0.301	β = **0.22**, *p* = **0.016**	β = −0.17, *p* = 0.081
PRS MDD 0.10	β = −0.09, *p* = 0.144	β = **0.25**, *p* = **0.012**	β = −0.19, *p* = 0.066
PRS MDD 0.20	β = −0.11, *p* = 0.084	β = **0**.**24**, *p* = **0**.**019**	β = −0.18, *p* = 0.085
PRS MDD 0.30	β = −0.10, *p* = 0.102	β = **0.24**, *p* = **0.018**	β = −0.19, *p* = 0.080
PRS MDD 0.40	β = −0.10, *p* = 0.107	β = **0**.**24**, *p* = **0**.**022**	β = −0.18, *p* = 0.092
PRS MDD 0.50	β = −0.11, *p* = 0.079	β = **0.25**, *p* = **0.014**	β = −0.20, *p* = 0.064

PRS MDD […] = polygenic risk score for major depression, calculated based on the mentioned *p-*value threshold. Significant interactions (*p* < 0.05) are in bold

^a^The average age of participants at the intercept was 13.00 years old (*SD* = 0.44)

^b^The PRS MDD 5 × 10^−8^ score was based on the meta-analysis by Hyde et al. (2016) and corrected for adolescent age, sex, and population stratification. The other 12 polygenic risk scores for major depression with different *p-*value thresholds were based on the 23andMe summary statistics and also corrected for adolescent age, sex, and population stratification

**Fig. 3:**
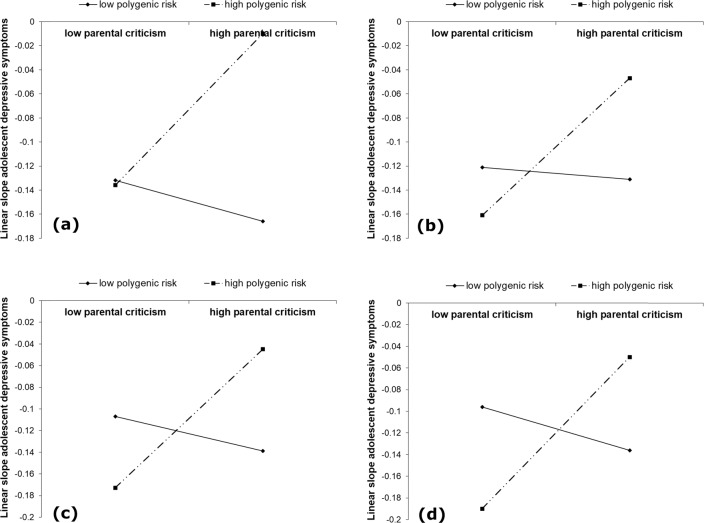
Graphical representation of the significant interactions in the sensitivity analyses between the corrected polygenic risk scores for major depression and the multi-informant longitudinal index of parental criticism on linear slope levels of adolescent depressive symptoms, for *p-*value thresholds (**a**) *p* < 5 × 10^−7^, (**b**) *p* < 5 × 10^−3^, (**c**) *p* < 0.05, and (**d**) *p* < 0.50

The interaction between the corrected polygenic risk score for major depression (i.e., genetic risk for major depression, as identified by Hyde et al. 2016) and parental criticism was not significantly associated with changes in adolescent depressive symptoms over time (i.e., the linear or the quadratic slope). However, results from the sensitivity analyses suggested that most of the 12 additional polygenic risk scores interacted significantly with parental criticism in predicting linear changes in adolescent depressive symptoms over time, βs = 0.11–0.25, *p*s = 0.007–0.184 (see Table 5). A visualization of predicted linear slope values of adolescent depressive symptoms at low (−1 SD) and high (+1 SD) levels of parental criticism polygenic risk for major depression suggested an interesting change in the pattern of interactions from the genetically-selective polygenic risk scores to the more genetically-inclusive polygenic risk scores (see Fig. 3). Specifically, for the genetically-selective polygenic risk scores (i.e., *p* < 5 × 10^−7^ and *p* < 5 × 10^−6^) lower levels of parental criticism were associated with lower slope levels of adolescent depressive symptoms, regardless of polygenic risk for major depression, whereas higher levels of parental criticism were associated with higher slope levels for adolescents with higher polygenic risk for major depression but lower slope levels for adolescents with lower polygenic risk for major depression (see Fig. 3a, b). In contrast, for the more genetically-inclusive polygenic risk scores (i.e., *p* < 0.05–*p* < 0.50) adolescents with high polygenic risk for major depression showed the lowest slope levels of adolescent depressive symptoms at low levels of parental criticism, but the highest slope levels at high levels of parental criticism (see Fig. 3c, d). Adolescents with low polygenic risk for major depression showed higher slope levels of adolescent depressive symptoms at low levels of parental criticism and lower slope levels at high levels of parental criticism (see Fig. 3c, d). Thus, for the more genetically-inclusive polygenic risk scores findings suggested a cross-over interaction.

The models with the exploratory adolescent sex × polygenic risk for major depression × parental criticism interactions failed to achieve acceptable fit to the data, and resulted in estimation problems in attempts to improve fit, and the model complexity exceeded this study’s sample size. Therefore, the results of these exploratory analyses were not reported.

## Discussion

Adolescence is a critical period for the development of depressive symptoms (Kessler et al. 2012) and research that focuses on identifying factors that affect this development is essential. The etiology of depression is complex (Garber and Rao 2014) and research has focused increasingly on how the interplay between genetics and environment (G × E) are associated with depression. Yet, G × E studies on depression are scarce in adolescence, particularly longitudinal studies that focus on depressive symptom development and that apply a polygenic approach. Therefore, this study aimed to examine (1) whether polygenic risk for major depression (as identified among adults of European descent in a recent meta-analysis; Hyde et al. 2016) was associated with adolescent depressive symptom development from early to late adolescence, and (2) whether polygenic risk for major depression moderated the effect of parental criticism on adolescent depressive symptom development from early to late adolescence.

The findings from the present longitudinal community study suggested that polygenic risk for major depression, as identified in a recent meta-analysis (Hyde et al. 2016), was associated with higher mean levels of depressive symptoms from early to late adolescence. Moreover, polygenic risk for major depression significantly moderated the association between parental criticism and mean levels of depressive symptoms from early to late adolescence. Specifically, as hypothesized, the highest mean levels of depressive symptoms across adolescence were found for those adolescents experiencing higher levels of parental criticism and with higher polygenic risk for major depression (i.e., genetically vulnerable individuals). Polygenic risk for major depression was not significantly associated with changes in adolescent depressive symptoms over time. Furthermore, sensitivity analyses suggested that that polygenic risk for major depression based on different *p* value thresholds was consistently associated with higher mean levels of depressive symptoms from early to late adolescence (see Table 3), suggesting robustness of the findings. Consistent with the main findings, polygenic risk for major depression did not consistently moderate the association between parental criticism and *mean levels* of depressive symptoms across adolescence in the sensitivity analyses. However, in contrast to the main analyses polygenic risk for major depression did consistently moderate the association between parental criticism and *change* in depressive symptoms across adolescence in the sensitivity analyses (see Table 5). Moreover, in these analyses the pattern of the interactions appeared to change from the genetically-selective polygenic risk scores to the more genetically-inclusive polygenic risk scores (see Fig. 3). In sum, findings in this study suggested that polygenic risk for major depression was associated with higher depressive symptoms from early to late adolescence, particularly for those adolescents experiencing high levels of critical parenting. In addition, the findings highlight that different indices of genetic risk, for example in the present study captured by different *p*-value thresholds, may show different G × E interaction patterns.

The main effect of polygenic risk for major depression in adulthood on adolescent depressive symptom development showed that information on polygenic risk for major depression, as identified among adults in a large-scale GWAS (Hyde et al. 2016), is also highly relevant for predicting depressive symptom development from early to late adolescence in the general population. This is in line with the developmental psychopathology perspective (Cicchetti and Rogosch 2002) and a dimensional view on psychopathology (e.g., Hankin et al. 2005), in which major depression represents the extreme end of a continuous distribution of symptom severity at the population level. The finding that adolescents with higher polygenic risk for major depression showed higher mean (i.e., intercept) levels of depressive symptoms across adolescence suggests that genetic risk for major depression in adulthood already expresses itself in the form of heightened levels of depressive symptoms throughout adolescence. Polygenic risk for major depression was not significantly associated with changes in adolescent depressive symptoms over time on top of higher mean levels of depressive symptoms. This pattern of findings was replicated in the sensitivity analyses. Also, there were no consistent sex differences in the association between polygenic risk for major depression and mean levels or changes of depressive symptoms across adolescence. Whereas some have suggested that the emergence of sex differences in depressive symptoms in adolescence (Hankin et al. 2015) may be explained by higher vulnerability to genetic risk for adolescent girls compared to boys (Scourfield et al. 2003), no consistent evidence for such sex differences were found in this study.

In addition to the significant main effect of polygenic risk for major depression on mean levels of depressive symptoms across adolescence, in the main analyses genetic risk for major depression was also found to moderate the association between parental criticism and mean levels of depressive symptoms across adolescence (i.e., G × E). As hypothesized, parental criticism was particularly associated with higher mean levels of depressive symptoms across adolescence for those adolescents with higher polygenic risk for major depression. At the same time, lower levels of parental criticism were associated with lower mean levels of adolescent depressive symptoms, regardless of polygenic risk for major depression (see Fig. 2). In line with the Diathesis–Stress framework (Monroe and Simons 1991), polygenic risk for major depression, captured by only those genetic variants that were found to reach genome-wide significance (*p* < 5 × 10^−8^) among adults in a recent meta-analysis (Hyde et al. 2016), thus appeared to distinguish adolescents based on vulnerability versus resilience. Specifically, the highest depressive symptoms across adolescence were found for those adolescents with both high levels of critical parenting and high polygenic risk for major depression (i.e., genetically vulnerable individuals), whereas mean levels of depressive symptoms across adolescence were not affected by critical parenting for adolescents with low polygenic risk for major depression (i.e., resilient individuals). In the sensitivity analyses, this significant G × E finding on mean levels of adolescent depressive symptoms was only replicated for the most genetically-selective polygenic risk score (i.e., *p* < 5 × 10^−7^), but not any of the other additional polygenic risk scores (see Table 5).

Whereas no significant G × E interaction was found in the prediction of changes in adolescent depressive symptoms over time in the main analyses, in the sensitivity analyses polygenic risk for major depression did quite consistently moderate the association between parental criticism and change in depressive symptoms across adolescence. Moreover, the pattern of the interactions appeared to change from the genetically-selective polygenic risk scores to the more genetically-inclusive polygenic risk scores (see Fig. 3). Specifically, for the genetically-selective polygenic risk scores (e.g., *p* < 5 × 10^−7^ and *p* < 5 × 10^−5^) low levels of parental criticism were associated with a (normative) decrease in adolescent depressive symptoms, regardless of genetic risk for major depression, whereas higher levels of parental criticism were associated with a weaker decrease (or rather, stability) in adolescent depressive symptoms for adolescents with higher compared to lower genetic risk for major depression. In contrast, for the more genetically-inclusive polygenic risk scores (i.e., *p* < 0.05–0.50) low levels of parental criticism were associated with a stronger decrease in adolescent depressive symptoms for adolescents with higher polygenic scores compared to adolescents with lower polygenic scores, whereas higher levels of parental criticism were associated with a weaker decrease (or rather, stability) in adolescent depressive symptoms for adolescents with higher polygenic scores compared to adolescents with lower polygenic scores (i.e., a cross-over interaction). Thereby, the interaction patterns appeared to change from being in line with the Diathesis–Stress framework (Monroe and Simons 1991) for the more genetically-selective polygenic risk scores, in line with the G × E finding on mean levels of adolescent depressive symptoms in the main analyses, to being in line with the Differential Susceptibility framework (Belsky et al. 2007; Belsky and Pluess 2009) for the more genetically-inclusive thresholds, although no formal statistical tests were conducted to distinguish the interaction effects in terms of Diathesis–Stress versus Differential Susceptibility (see Roisman et al. 2012).

In terms of “substantive meaning”, genetically-selective polygenic risk scores are based on a few SNPs that are strongly significantly associated with the specific phenotype (e.g., *p* < 5 × 10^−8^). So, these polygenic risk scores likely represent genetic risk scores for a specific phenotype, such as (self-reported) major depression in this study, and appear to distinguish individuals based on environmental vulnerability versus resilience (in line with a Diathesis–Stress framework; Monroe and Simons 1991). In contrast, genetically-inclusive polygenic risk scores are based on thousands of SNPs across the genome with increasingly weaker associations with the specific phenotype, but thereby better reflecting the total SNP-heritability of major depression. In this study, these polygenic risk scores appeared to distinguish individuals based on environmental plasticity with the highest depressive symptoms for those adolescents with high polygenic risk at high levels of critical parenting, but also the lowest depressive symptoms at low levels of critical parenting (in line with the Differential Susceptibility framework; Belsky et al. 2007; Belsky and Pluess 2009). Importantly, past G × E interaction research on (adolescent) depressive symptoms has found evidence for findings in line with both Diathesis-Stress and Differential Susceptibility, and the present study highlights that different G × E interaction patterns may be found depending on the genetic index that is created (e.g., the applied *p-*value threshold for calculating a polygenic risk score).

Although it was not the main focus of this study, it is worth noting that correlations between the environmental factor parental criticism and the polygenic risk scores for MDD appeared to increase in strength with more inclusive *p-*value thresholds, particularly for those thresholds higher than 0.01 (see descriptive statistics in Table 1). As the more genetically-inclusive polygenic risk scores include more SNPs across the genome that are less strongly associated with the specific phenotype of interest, (self-reported) major depression in this study, these scores may include SNPs that are not uniquely associated with the phenotype of interest but may be associated with other phenotypes as well. Combined with the observation of stronger correlations between parental criticism and the more genetically-inclusive polygenic risk scores for MDD, one might question that these scores somehow reflect gene-environment correlation (rGE) associated with MDD either in itself or in the G × E interactions. However, such rGE is not a likely explanation. First, polygenic risk scores with more inclusive *p* value thresholds more closely approximate the total SNP-heritability of the phenotype of interest, that is, the proportion of variation in a phenotype that can be explained by additive effects of observed commonly-occurring genetic variants or SNPs (h^2^_SNP_). While more inclusive *p*-value thresholds might be more likely to include some “environmentally sensitive” SNPs that may confound the polygenic risk scores’ reflection of the total heritability of a phenotype (h^2^), this is not the same as capturing rGE. Second, the G × E analyses explicitly included the correlation between the polygenic risk scores and the environmental factor. Thereby, any potential environmental “confounding” in the G × E interactions is corrected for in these analyses and the polygenic risk scores’ explained variance would “purely” reflect the proportion of SNP heritability defined by the *p*-value threshold. It may, however, be an interesting direction for future research to systematically investigate whether more genetically-inclusive polygenic risk scores for MDD are more strongly associated with different relevant environmental factors compared to more genetically-selective polygenic risk scores and whether this appears to be the case across other phenotypes as well, to examine whether this phenomenon can be more widely observed than this study.

### Implications

The fundamental knowledge resulting from this study on G × E interactions has some interesting implications, although cautious interpretation is warranted considering issues such as sample size and generalization (which is further elaborated upon below). First, it adds to the etiological literature (Garber and Rao 2014) on individual differences in (a) vulnerability to develop depressive symptoms (i.e., significant genetic and environmental main effects), and (b) vulnerability/sensitivity to environmental exposure related to depressive symptom development (i.e., significant G × E interactions). Another finding of note in this respect is the relevance of parenting as a “general” measure of environmental exposure. Whereas past research has often focused on “high impact” environmental factors, such as child maltreatment or abuse or traumatic/stressful life events (Dunn et al. 2011), this study found that normal variation in parenting experienced by adolescents from the normal population also has the potential to interact with (i.e., exaggerate or attenuate) genetic risk in predicting depressive symptom development across adolescence. So, in addition to a focus on such “high impact” environmental factors in clinical practice, normal variation in environmental experiences (such as parenting) may deserve explicit attention.

Second, this knowledge is relevant in the context of personalized medicine and therapygenetics (see discussion in e.g., Lester and Eley 2013). Therapygenetics refers to the prediction of psychological therapy outcomes from genetic markers. In this context, knowledge on adolescents’ genetic susceptibility to the environment could potentially be used to predict their treatment response. Whereas genetically susceptible adolescents could potentially benefit from a brief and mild intervention, less susceptible adolescents might need a more intensive and comprehensive intervention to reduce depressive symptoms. Alternatively, genetic variation might differentially predict response to specific interventions, such as Cognitive Behavioral Therapy versus pharmacological treatment (e.g., Selective Serontonin Reuptake Inhibitors [SSRIs]). Within the broader context of personalized medicine, a fuller and more nuanced understanding of both genetic vulnerability and genetic sensitivity to the environment—or the developmental psychopathology of different depression more generally—might eventually aid in decisions on which treatment to select to maximize the chance of recovery for a particular individual. Even though personalized medicine concerning psychopathological treatment and therapygenetics are still in their infancy, in the future they may prove to be of great importance in guiding treatment selection and improving treatment effectiveness and outcome which, in turn, alleviates psychopathological symptoms and associated impaired functioning in many domains (Beevers and McGeary 2012).

### Strengths, Limitations, and Directions for Future Research

This study has several strengths. First, the main analyses relied on information from a recent meta-analysis (Hyde et al. 2016), which includes the first robust findings concerning polygenic risk for major depression (identified through self-report questionnaires) in individuals of European descent based on GWAS. Moreover, this information was complemented with information from the large 23andMe dataset to address issues of replication, sensitivity, and robustness within this study’s unique multi-informant longitudinal community sample. Second, by interacting polygenic risk for major depression with a multi-informant longitudinal index of critical parenting, this study followed recent recommendations for G × E studies to include polygenic approaches (Belsky and Israel 2014; Dick et al. 2015) and to increase the assessment quality of environmental exposure to increase power (Wong et al. 2003). Third and final, this study’s 6-year longitudinal design captures an extended period from early to late adolescence and thereby offers critical information on potential predictors of depressive symptom development in a developmentally vulnerable period (Garber and Rao 2014), which is scarce as most G × E research has been cross-sectional in nature and conducted in adult samples. Thereby, this study’s data-inferred polygenic developmental psychopathological perspective on the study of genetic main effects and G × E interaction effects on adolescent depression addresses several gaps in the literature.

At the same time, this study should be considered in the light of some limitations, which may provide potential alternative explanations for adolescent depressive symptom development within a multifactorial developmental psychopathology framework (e.g., equifinality; Cicchetti and Rogosch 2002) as well as directions for future research. First, while within the current sample the results were replicated across several subsequent polygenic risk score *p* value thresholds, suggesting robustness of findings in this sample, these analyses do not preclude the need for independent replications as with all G × E analyses. Also, while this study represents a unique polygenic, longitudinal G × E interaction approach on adolescent depressive symptom development that provides important preliminary insights, cautious interpretation and replication of findings in much larger samples is warranted given the relatively small sample size. On a related note, caution should be taken in generalizing the findings from this study to other populations and situations. The present sample consists of a relatively well-functioning community sample of adolescents with a relatively homogeneous ethnic background from one country in Europe and it is unclear whether the present results can be extended to adolescents from other regions of the world, who have a more diverse socio-economic and ethnic background, and who are more diverse in functioning. Furthermore, future research should expand on this study focusing on other relevant parenting, or more broadly, other relevant environmental, variables. Also, testing the exploratory adolescent sex × polygenic risk for major depression × parental criticism interactions was unfortunately not possible in this study, for which larger longitudinal samples are required. It has also been suggested that genetic risk for major depression may interact in sex-specific ways with “interpersonal stressors” other than those in the parental environment, such as peer stressors, in predicting depressive symptom development (Oldehinkel and Bouma 2011; Shih et al. 2006).

Second, PRSs for major depression are purely “descriptive” in a way that they only represent a summary measure of genetic risk for major depression by the number of risk alleles associated with major depression as identified in GWAS and do not inform in any way on potential biological mechanisms underlying genetic risk. Thereby, all significant G and G × E findings are mere statistical results that are in need of an explanation in terms of their underlying (biological) mechanisms. On a related note, the field is currently rapidly evolving with new genome-wide association meta-analyses for different phenotypes being published more commonly, including major depression (e.g., Wray et al. 2018). This study has specifically relied on a recent meta-analysis (Hyde et al. 2016) and the 23andMe dataset for the polygenic risk score calculations, because they relied on self-reports for identification of major depression case status. Since this study also relied on self-reports of depressive symptoms, this meta-analysis (Hyde et al. 2016) and the 23andMe dataset more closely match this sample than other studies, which may enhance the relevance of these genetic studies to the present study (although strong positive genetic correlations have been found between the 23andMe dataset and carefully curated clinical samples; Hyde et al. 2016; Wray et al. 2018).

Third and final, this study incorporated a longitudinal multi-informant average of critical parenting from early to late adolescence because of reasons of model complexity. Specifically, with 32 free parameters estimated in the current G × E analyses and a sample size of 327, the analyses were at the recommended number of cases to the number of free parameters ratio of 10:1 (Kline 2005, p. 111). Including a more complex representation of parental criticism in the G × E analyses would increase the number of free parameters and thereby bring the ratio cases/parameter ratio under the recommended 10:1, implying that model complexity would exceed the study’s sample size. An important challenge for future research would be to incorporate longitudinal assessments of the environmental factor included in G × E, in addition to longitudinal assessments of the phenotype of interest, because environmental factors such as parenting are dynamic in nature and may thereby show change, as well as differential effects, over time. Larger sample sizes are needed to, for example, include a latent longitudinal multi-informant index of parenting, in which each wave of each informant would be differentially weighted in contributing to the overall latent construct, or a Latent Growth Curve Model of (multi-informant) parenting, in which initial levels and change over time of the environmental factor are associated with those of the phenotype of interest and these associations would be moderated by genetic risk. Also, although the correlation between the genetic risk scores and critical parenting was included in all models to account for potential gene-environment correlations, potential intergenerational transmission of genetic risk must be acknowledged. This could either be direct transmission of genetic variation associated with depressive symptoms from parents to their adolescents or indirect transmission, as critical parenting can be rooted in depressive (or other psychiatric) symptoms in parents and thereby predict adolescents’ depressive symptom development (i.e., mediation processes from G to E rather than moderation of G × E; Harold et al. 2011; McAdams et al. 2014).

## Conclusion

While research has focused increasingly on the interplay between genetics and environment (G × E), studies in adolescence are scarce and few have used a longitudinal design to consider the developmental psychopathological properties of depression. Moreover, only few studies have applied a polygenic approach in gene-by-environment interaction studies. Therefore, this longitudinal community study examined the interaction between polygenic risk for major depression and critical parenting in relation to depressive symptom development from early to late adolescence. First, this study showed that polygenic risk for major depression is robustly associated with higher depressive symptoms across adolescence. Second, polygenic risk for major depression interacted with critical parenting in predicting depressive symptom development across adolescence, with the highest levels of depressive symptoms across adolescence for high polygenic risk combined with high levels of critical parenting (in line with the Diathesis–Stress framework; Monroe and Simons 1991). Overall, the results highlight how polygenic risk for major depression in combination with a general environmental factor impacts adolescent depressive symptoms from early to late adolescence. In addition, this study illustrates that different indices of genetic risk, e.g., by relying on different *p* value thresholds for their calculation, may show different G × E interaction patterns.

## Footnotes

1 SNP-heritability = the maximum proportion of variation in the phenotype that can be explained by additive effects of SNPs.

2 Participants were asked to complete the questionnaires around the same months every year for 6 successive years (January–March) and their age therefore increased by almost exactly 1 year every wave.

3 Full GWAS statistics could only be requested for the 23andMe discovery dataset (for more information, please see the paragraph on “Data access” in the Online Methods of Hyde et al. 2016) and were not available for the joint meta-analysis of the 23andMe discovery dataset, the Psychiatric Genomics Consortium dataset (including the Psychiatric Genomics Consortium MDD, bipolar disorder, SCZ1, SCZ1 + SWE, and the SCZ2 datasets), and the 23andMe replication dataset. Importantly, the 23andMe discovery dataset represented the largest sample size within the joint analysis (75,607 cases of depression and 231,747 controls) compared to the two other included datasets (9240 cases and 9519 controls for the Psychiatric Genomics Consortium dataset and 45,773 cases and 106,354 controls for the 23andMe replication dataset, respectively). Specifically, the 23andMe discovery dataset consists of 62% females with a European ancestry, with the majority (more than 87%) being 30 years or older (for more details, see Hyde et al. 2016).

4 Sensitivity analyses suggested that polygenic risk scores controlled for all 20 Dutch principal components resulting from PLINK’s Principal Components Analysis were strongly correlated with the polygenic risk scores controlled only for the top 5 Dutch principal components, *r*s ≥ 0.958, *p*s < 0.001.

5 Because sex differences in adolescent depressive symptoms have been robustly reported (e.g., Hankin et al. 2015), sex was included as a covariate in all analyses. Excluding sex as covariate from all analyses did not affect any of the main conclusions.
